# The Effect of Alpha-Cyclodextrin on Postprandial Glucose Excursions: a Systematic Meta-Analysis

**DOI:** 10.7759/cureus.31160

**Published:** 2022-11-06

**Authors:** Knut M Wittkowski

**Affiliations:** 1 Research and Development, ASDERA LLC, New York, USA

**Keywords:** high carbohydrate, postprandial blood glucose (ppbg), dietary fiber, insulin, dietary supplement

## Abstract

Alpha-cyclodextrin (αCD) is a bacterial product that is widely used as a food ingredient. In the European Union (EU), αCD is regulated as a dietary fiber with an authorized health claim “for contributing to the reduction of postprandial glycemic responses.” In the US, αCD is generally recognized as save (GRAS), but on April 25, 2022, the U.S. Food and Drug Administration (FDA) rejected the inclusion of αCD in the list of dietary fibers because “the strength of the scientific evidence does not support a finding of a beneficial effect of αCD on postprandial blood glucose …” To evaluate the strength of this scientific evidence, this meta-analysis reviews clinical trials conducted to test the effect of αCD on the rise of blood glucose and insulin levels during three hours after consumption of a meal comprising carbohydrates, fats, and proteins.

Several issues related to the standardization of the outcomes, the choice of the statistical methods in the cross-over studies conducted, and the choice of methods for the aggregation of P-values are discussed. It is concluded that the administration of αCD not only reduces the postprandial glycemic responses, but the absence of an increase in insulin levels suggests that αCD acts independently of increasing insulin production and, thus, the beneficial effect of αCD is not affected by insulin resistance.

## Introduction and background

As soluble fibers, cyclodextrins (CDs) comprise several glucose molecules. In contrast to many other resistant fibers, however, CDs form rings, rather than linear structures, which makes them a prebiotic accessible only to some (commensal) bacteria that have the enzymes to open these rings so that they can digest the glucose units.

These rings have an interesting physicochemical property: they are hydrophilic on the outside, making the CDs water-soluble, and lipophilic on the inside, allowing them to carry lipids through water (gut, serum), either to deliver lipid drugs or to filter out (“deplete”) lipids.

Alpha-, beta-, and gamma-CDs (αCDs, βCDs, γCDs) are rings of six, seven, and eight sugars, respectively, and, thus, can fit lipids of different sizes. αCDs bind preferentially saturated and trans fatty acids (FAs) [1-5]. The larger βCDs can also fit steroids and sterols while γCDs are large enough to carry even larger molecules. Their high specificity for “bad” FAs makes αCDs particularly interesting as dietary fibers.

This meta-analysis aims to integrate the information from these clinical trials assessing the effect of αCDs to reduce the increase of blood sugar after a carbohydrate (CHO)-rich meal. using standardized proportions of CD vs CHO or fat contained in the meals and standard statistical methodology for combining P-values in meta-analyses.

A previous version of this article was posted to the medRxiv preprint server on September 6, 2022 [6].

## Review

Methods

Systematic Review and Meta-Analysis

Guidelines: This systematic review and meta-analysis was carried out in accordance with guidelines issued by Preferred Reporting for Systematic Reviews and Meta-analyses (PRISMA) [7].

Study selection: All English language articles identified from three databases (see Materials) were imported into one Endnote library where all duplicates were removed. Studies that did not report the required data were excluded.

Data extraction: For each included article, the means, standard errors, and P-values related to the incremental area under the curve (iAUC) were extracted as reported. Other extracted variables were age, gender, study duration, year of publication, and methodological characteristics.

Statistics

Fisher’s combined probability test (FCPT) [8] is the most asymptotically optimal method in terms of Bahadur efficiency for aggregating information from P-values [9]. If results are concurrent (pointing in the same direction), the combined P-value can be calculated from:

2 Σ_i=1…k_ −ln(P_i_) ~ χ^2^_2k_

For instance, the requirement of two studies (k = 2) significant at the conventional P_i _= 0.05 level for drug approval translates to a combined P(4×2.996 = 11.98 > χ^2^_4_) = .0175. One important consequence is that including an additional concurrent test with P < 0.37 (asymptotically) improves overall significance [10]. Hence, a third study with any P = 0.05 … 0.37 improves the overall significance of two studies conventionally significant at the 0.05 level each, rather than raising questions about their overall significance. If one of the two studies were significant at a level of e.g., P < .01, the third study might have to point in the opposite direction to void the significance of the other two studies.

When P-values are reported as categorial (P > 0.05), a meta-analysis can also be guided by the inspection of the figures in the source publications. To facilitate comparisons, the figures given below have been scaled and cropped, and elements have been resized or removed (like indicators of significance of comparisons not being considered) while some information has been added from the text. No data were removed, except values beyond 180 min (3 h) in two studies [4,11].

Materials

Literature Search

In September 2022, three publicly available databases (clinicaltrials.gov, PubMed, and Google Scholar) were searched for clinical trials regarding the efficacy of αCD in reducing postprandial glucose (PPG) excursions after a meal rich in CHOs. A manual search of the reference lists of included articles and their citations was conducted to identify any articles not retrieved by the database searches.

Inclusion and Exclusion Criteria

This systematic review included clinical studies of dietary interventions that (1) enrolled generally healthy subjects regardless of their age and background, (2) were randomized or non-randomized, (3) included CHOs in the meals, (4) included αCD as an intervention, and (5) included PPG blood profiles as an outcome. Several publications also included insulin and triglyceride (TG) profiles, but a search for “insulin” or “triglyceride” did not yield additional results.

Records Retrieved

**Figure 1:**
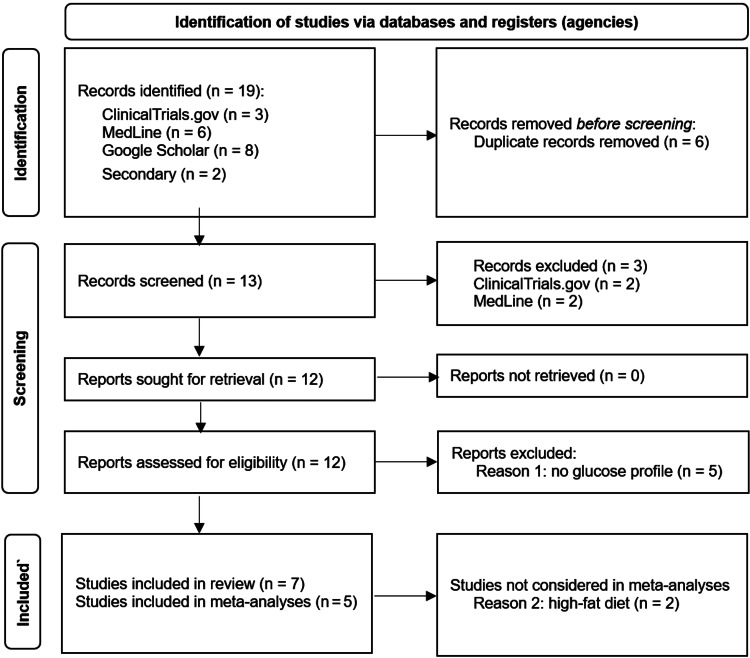
PRISMA flow diagram Preferred Reporting Items for Systematic Reviews and Meta-Analyses (PRISMA) 2020 [7]

Data sources used were the above databases as well as the publications identified as relevant in these sources or referencing the sources (Table 1). The PRISMA 2020 flow diagram is shown in Figure 1.

**Table 1:** Records retrieved by source Records included: *, excluded: -, identified in references or citations: »

Source/ Reference	(Search strategy: query terms) / Comments
CLINICALTRIALS.GOV	(Study type: Interventional Studies (Clinical Trials), Intervention/Treatment: Alpha-Cyclodextrin, Outcome Measure: Glucose, Other term: carbohydrate – removed to get results)
* Lytle, … Jensen (2018) [11]	NCT02999620 / NCT03002168
- Amar et al. (2016) [12]	NCT01131299 – 6 g/d, 12–14 wk, no single-day profiles taken
- Soldavini et al. (2022) [13]	NCT05393843 – terminated because of poor compliance because of coronavirus disease 2019 (COVID)
MEDLINE	(Clinical Trial, “carbohydrate alpha-cyclodextrin glucose”)
* Buckley et al. (2006) [14]	
- Comerford et al. (2011) [15]	– glucose profiles not an outcome
» Grunberger et al. (2007) [16]	– serum glucose in diabetic patients not reported
* Gentilcore et al. (2011) [4]	
* Jarosz, Flexner et al. (2013) [17]	
- Amar et al. (2016)	– duplicate
- Bessell et al. (2020) [3]	– glucose profiles not an outcome, deviation from study protocol (“dietary advice for weight loss”) [18]
GOOGLE SCHOLAR	(carbohydrate alpha-cyclodextrin glucose postprandial clinical)
- Buckley et al. (2006)	– duplicate
- Gentilcore et al. (2011) [4]	– duplicate
- Jarosz, Fletcher, et al. (2013)	– duplicate
- Amar (2016) et al.	– duplicate
- Jain et al. (2016)	– glucose profiles are not an outcome
* Bär, Diamantis, et al. (2020) [5]	– in PubMed, but not as a “clinical trial”, unpublished protocol (2002) and report (2002) quoted in EFSA (2012) [19] some results also described in Schmid (2004) US 2004/0161526 A1 (Wacker, abandoned) [20]
» Sugahara, Inoue, et al. (2016) [21]	– not in PubMed
- Lytle, …, Jensen (2018)	– duplicate
* Binou et al. (2022)	– NCT04725955, “enriched wheat bread” listed as treatment – not in PubMed

The European Food Safety Authority (EFSA) based its 2012 scientific opinion on three clinical trials (Table 1). The U.S. Food and Drug Administration (FDA) included two more recent studies, where the meals, in particular in the former study, also contained substantial amounts of fat and protein. Three additional publications were identified in this review. One had glucose profiles only as a secondary outcome, the other two were published only after the FDA had made its decision, one is included in the meta-analysis, another study (a combination treatment with 5 g αCD per meal, 45% calories from CHOs) was terminated because of poor compliance during COVID [11,13,22,23].

Results

Data Extraction

**Figure 2:**
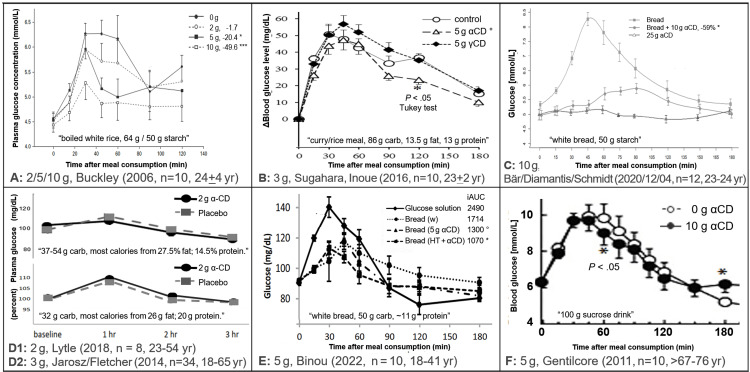
Data extraction summary: effects of different doses of αCD on blood glucose profiles. In all studies, each of >8 patients was administered all doses (cross-over). Legend: ***: P < .001; *: P < .05; °: P < .37, Figures were derived (excerpted) under 17 U.S.C. § 107 (fair use in scholarship and research) from Buckley, et al. (2006, permission granted) [14], Sugahara, Inoue, et al. (2016) [21], Bär, Diamantis, et al. (2020, CC BY 4.0) [5] Fletcher, et al. (2014, dissertation) [17], Lytle, et al. (2018, redrawn) [11], Binou, et al. (2022, permission granted) [22,23], Gentilcore, et al. (2011, permission granted) [4]: To serve the purpose of scholarly criticism, figures have been scaled to have similar axes, scales were cropped at 180 min [4,11], colors and some misleading indicators of “significance” were removed, and information was added to their sub-legends. αCD: alpha-cyclodextrin

**Figure 3:**
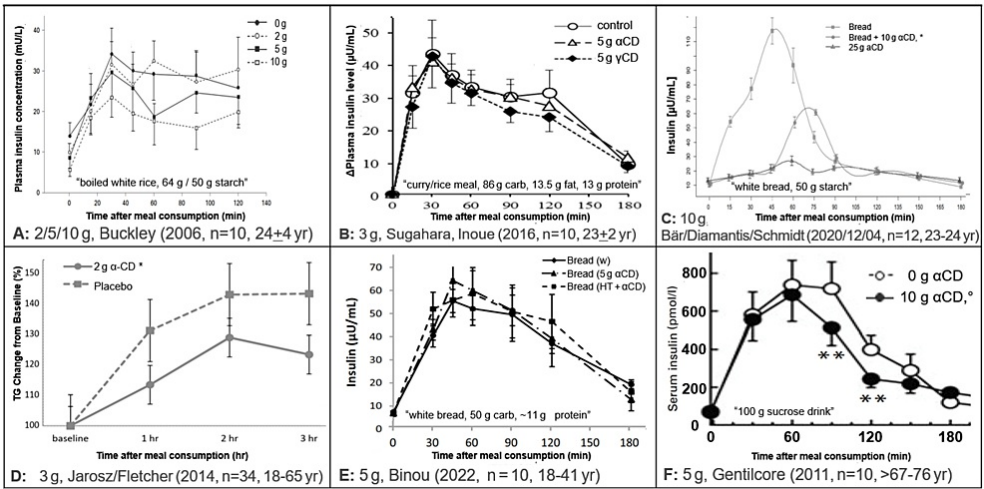
Data extraction summary: effects of different doses of αCD on serum insulin and TG Legend: ***: P < .001; *: P < .05; °: P < .37, figures were derived (excerpted) under 17 U.S.C. § 107 (fair use in scholarship and research) from Buckley, et al. (2006, permission granted) [14], Sugahara, Inoue, et al. (2016) [21], Bär, Diamantis, et al. (2020, CC BY 4.0) [5] Fletcher, et al. (2014, dissertation) [17], Lytle, et al. (2018, redrawn) [11], Binou, et al. (2022, permission granted) [22,23], Gentilcore, et al. (2011, permission granted) [4]: To serve the purpose of scholarly criticism, figures have been scaled to have similar axes, scales were cropped at 180 min [4,11], colors and some misleading indicators of “significance” were removed, and information was added to their sub-legends. αCD: alpha-cyclodextrin; TG: triglyceride

Table 2 shows the numerical results extracted from the seven studies included in the review. Figure 2 shows the average glucose profile data by diet as presented in the seven clinical trials. Figure 3 shows the serum insulin or triglyceride (TG) profiles as provided in the publications. Table 3 presents a summary of the data.

**Table 2:** Characteristics of studies previously and presently reviewed FDA: U.S. Food and Drug Administration (FDA); EFSA: European Food Safety Authority; CHO: carbohydrate; αCD: alpha-cyclodextrin; PPG: postprandial glucose; TG: triglyceride

Review	Study	Characteristics
EFSA	Buckley et al. (2006) [14]	10 healthy adults aged 24 ± 4 yr consumed boiled rice (50 g digestible CHOs) with 0, 2, 5, or 10 g of added αCD. PPG and insulin were assessed.
	Diamantis, Bär [5]	unpublished 2002 protocol and report, quoted in EFSA (2012) [19], subsequently published by Bär, Diamantis, et al. (2020) [5]): 12 healthy male adults aged 23–24 consumed white bread (50 g starch) with 0 or 10 g αCD dissolved in 250 mL drinking water. PPG and insulin were measured. The study was also included in the patent application Schmid (2004) [20]
	Gentilcore et al. (2011) [4]	10 healthy older subjects aged 68–78 yr consumed 100 g sucrose with 0 or 10 g αCD dissolved in water. PPG and insulin were measured.
..., FDA	Jarosz, Fletcher, et al. (2013) [24]	Dissertation (Fletcher 2013) [24] published subsequently by Jarosz et al. (2013) [17]: 34 healthy adults aged 18–65 yr consumed a commercially prepared egg sausage biscuit sandwich (32 g CHOs, 26 g fat, 20 g protein) with containing 0 or 2 g of αCD (2 pills). PPG and TG were measured.
	Sugahara, Inoue, et al. (2016) [21]	10 adults aged 22.9±1.8 consumed a beef curry and rice meal (86 g CHOs, 13.5 g fat, 11 g protein) with 0 or 5 g αCD. PPG, TG, and insulin were measured.
..., present	Lytle, …, Jensen, et al. (2018) [11]	8 healthy adults aged 23–54 yr consumed 2 g αCD with a liquid meal breakfast comprising 60% (37–54 g) CHOs, 27.5% fat, and 14.5% protein. While the primary outcome was fat, PPG was also measured.
	Binou et al. (2022) [22, 23]	10 healthy adults aged 18–41 (28.2±6.8) consumed white wheat bread (50 g CHOs, 2-4 g fat, 10–12 g protein) with 0 or 5 g of αCD. PPG and insulin were measured.

**Table 3:** Summary data … / 50 g: ... αCD [g] / 50 g CHO, ↓: visibly lower, ↔: visibly indistinguishable, ↑: visibly higher; FDA: U.S. Food and Drug Administration (FDA), EFSA: European Food Safety Authority, CHO: carbohydrate, αCD: alpha-cyclodextrin, TG: triglyceride

Agency	Publications	Subjects	Age [yr]	Ingredients	Source	αCD (g) (… / 50 g)	glucose (*P)*	insulin (*P*)	TG
EFSA,	Buckley et al. (2006) [14]	10	24 ± 4	50 g starch	white rice	2 (2)	↓ n.s.	↑ n.s.	
FDA	…	healthy	…	…	…	5 (5)	↓ .03	↔	
…	…	adults	…	…	…	10 (10)	↓ .001	↓ n.s.	
EFSA, FDA	Diamantis, Bär (2002a/b referred to in EFSA 2012) [19], Schmid (2004) [20], Bär, Diamantis, et al. (2020) [5]	12 healthy male adults	23–24	50 g starch / ~1 mL olive oil	white bread	10 (10)	↓ <0.05	↓ <0.05​​​	
	Binou et al. (2022) [22, 23]	10 healthy adults	18–41	50 g CHO / ~3 g fat / ~11 g protein	white wheat bread	5 (5)	↓ <0.06	↔	
EFSA, FDA	Gentilcore et al. (2011) [4]	10 healthy seniors	68–78	100 g sucrose		10 (5)	↓ n.s. (1 h: <0.05)	↓ 0.09 (2h: <0.001)	
FDA	Sugahara et al. (2016) [21]	10 healthy adults	23 ± 2	86 g CHO / 13.5 g fat / 11 g protein	beef curry with rice	5 (3)	↓ <0.37	↓ n.s.	↓ n.s.
FDA	Fletcher (2013), Jarosz et al. (2013) [17, 24]	34 healthy adults	18–65	32 g CHO / 26 g fat / 20 g protein	McMuffin egg sausage biscuit	2 (3)	↓ n.s.		↓
	Lytle, …, Jensen et al. (2018) [11]	8 healthy adults	23–54	~45 g CHO, ~21 g fat ~11 g protein	Ensure Plus liquid meal	2 (2)	↔		↔

Risk of Bias in Studies

Three studies were all supported by Wacker Chemie AG, Germany [4,5,14]. As stated, the company had no role in the design, execution, data analysis, and reporting of the data. Some of the authors of two studies were principals and employees of ArtJen Complexus, USA [24] and Cyclochem, Japan [21]. The authors declared no conflict of interest. Three studies [11,22,23] do not show any risk of potential conflict of interest. There is also no indication of missing results (reporting bias).

Heterogeneity Among Studies

All five studies included in the meta-analysis had a cross-over design and the sample size was 8-12 subjects. All studies observed subjects for at least 2 hr. The heterogeneity of meals was considered by standardizing the αCD/CHO ratio.

Data Review

One of the most striking observations is the glucose curves in the two studies with the highest fat and protein content [11,17]. where blood glucose levels increased by only 10% under control conditions as compared to ~50% in studies serving a high CHO diet (Figure 1D has been rescaled from [24] and Figure 2 to reflect that aspect). If a diet does not increase blood glucose sufficiently, the addition of αCD cannot have a significant effect, especially not in a small sample size. Hence, these studies were not considered for meta-analysis.

Since the earliest study [14], the use of standard error of the mean (SEM) bars shown in the graphs points to another, potentially common problem in this field: the data collected under different conditions seem to have been analyzed by statistical methods that ignore the within-subject correlations (WSC), which is a key element in the analysis of a cross-over design. Ignoring the WSC seems to have contributed to several of the results being reported as “non-significant” despite obvious differences seen in the figures presented. The text in another study [21], for instance, reads “the cumulative (incremental area under the curve) iAUC … was smaller in the αCD group … than in the control group.” This strongly suggests that the results are described as if the data were coming from different “groups,” so the WSC was ignored, diminishing the reported level of significance. In the latter study, their Figure 3 caption (here, Figure 2B) notes that “Tukey’s test” was used. Assuming that this refers to Tukey’s (1949) test for “comparing individual means in the analysis of variance” [25], this test is not appropriate for comparing the 0-180 min cumulative iAUC.

Meta-Analysis

Among the studies with high levels of CHO (bread and rice), the earliest study [14] (Figure 1A) noted “a dose-dependent inhibition of the (PPG) response to a standard [COH] meal following incorporation of (αCD)." The mean iAUC under the plasma glucose curve was negatively related to the dose of α-CD (r^2^ = 0.97, P = 0.02), with the iAUCs being significantly lower than under the control dose (0 g αCD) for the 5 h (P = 0.03) and 10 g (P = 0.001) doses.

As shown in Table 4, the comparisons based on the four subsequent studies with high-COH diets [4,5,21-23] are consistent with the dose-response relationship seen in the initial conclusion by the EFMA [14]. The two studies serving high-fat meals [11,17], instead, showed only a minor PPG excursion under the control dose and, thus, not even a small reduction of a PPG excursion under the low 2 g α-CD dose.

**Table 4:** Summary conclusions CHO: carbohydrate; αCD: alpha-cyclodextrin; TG: triglyceride; FCPT: Fisher’s combined probability test

αCD/CHO	Glucose	Glu	Other
2 g / 50 g	Not significant in the initial study [14] (one excluded study [11] was also ns)	↓ ns	Ins: ↑ ns
	No evidence for Glu/Ins efficacy		
3 g / 50 g	An obvious difference in Fig. 3 of [21] (5 g / 86 g) labeled as “not significant” suggests one of the statistical flaws identified.	↓ <0.37	Ins: ↓ ns
	McMuffin study [17, 24] excluded from the Glu meta-analysis is significant for TG		TG: ↓ <0.05
	Unclear evidence for Glu/Ins efficacy and TG efficacy		
5 g / 50 g	In the initial [14] and in a second study [22,23], 5 g / 50 g CHO were significant for Glu (at P = 0.03 and P = 0.06, respectively, combined P = 0.013 [8], well below the FCPT cut-off of 0.0175)	↓ <0.013	Ins: ↔ ns
	Including the 10 g αCD / 100 g sucrose study (P < .37 between "groups") [4] would add an Ins benefit but would not change the Glu conclusion		Ins: ↓ 0.09
	Significant evidence for Glu efficacy, some evidence for Ins efficacy		
10g / 50 g	In the initial [14] and in a second study [5] 10 g / 50 g CHO were significant for Glu/Ins (P < 0.001 and P < 0.05, respectively, combined P < 0.000055) [8]. One of them [5] was also significant for insulin.	↓ <0.001	Ins: ↓<0.05
	Highly significant evidence for Glu/Ins and significant evidence for TG efficacy		

In summary, all comparisons, even at the 2 g dose, showed lower glucose profiles with αCD and only one comparison (at the 2 g dose) [4] showed higher (but not significantly higher) insulin levels with αCD. Hence, the efficacy of αCD when added to a high CHO meal (rice or bread) among young adults is dose-dependent [19]; doses from 5 g/50 g are proven effective, as concluded by the EFSA in 2012. αCD’s mechanism of action seems not to involve an increase in insulin production. While αCD lowered TG in one high-fat study, more studies on the effect of αCD on blood lipids in humans need to be done.

Discussion

This meta-analysis addresses one of the hypothetical benefits of αCD: the reduction of PPG responses, where EFSA [19] and FDA [26] have come to different conclusions, even though they agree on the interpretation of the three studies available to the EFSA in 2012: (1) 50 g starch in white rice, 2/5/10 g αCD, young adults [14]: “a dose-dependent effect” [19], with no significant effect at 2 g, but “significant effects of 5 g and 10 g” [26]. (2): 50 g starch in white bread, 10 g αCD, young adults [5]: a (significant) effect [19,26]. (3) 100 g sucrose drink, 10 g αCD, seniors [4]: no effect [19,26].

EFSA

*“The Panel considers that the following wording reflects the scientific evidence: “Consumption of [αCD] contributes to the reduction of the blood glucose rise after starch-containing meals [and] in order to obtain the claimed effect, at least 5 g of [αCD] per 50 g of starch should be consumed”* [19].* *-- Two additional studies had been published at the time of the FDA’s review (see Materials) [26]. (1) High-fat meal with 32 g starch, 2 g αCD, adults [17]: no significant difference [26]. This study was excluded from the meta-analysis, because of the diet’s low CHO content. After this high-fat meal, however, αCD reduced TG. (2) Beef curry/rice meal, 86 g starch, 5 g αCD, adults [21], (2.9 g per 50 g of starch) no statistical difference [26]. Aside from a low dose of αCD, this study raises substantial issues regarding the validity of the statistical analysis, as discussed above.

FDA

*“There is inconsistent evidence … which weakens our confidence … ”* [26]. An additional informative study has been published since where 50 g starch in white bread was taken with 5 g αCD, yielding P = 0.06 (< 0.37) [22,23]. Despite being “not significant” on its own, this study clearly adds further evidence for the dose of 5 g αCD, when added to a high CHO, being effective. From the details provided in the FDA's assessment, there are several aspects in the published evidence that allow one to resolve at least some of the perceived inconsistencies.

Lack of Significance

In a meta-analysis, a “non-significant” result should not be interpreted as evidence against an effect (“Lack of proof of an effect is not proof of lack of an effect”). In particular, having one of several studies not reaching the conventional level of significance (P < 0.05) does not, in itself, create an inconsistency. With the FCPT [8], adding a study with P < 0.37 [21-23] typically suffices to strengthen the overall significance [10].

Within-Subject Correlation

Cross-over studies often have more power than two-group comparisons because data coming from the same person tend to be correlated. At least one of the studies [21], however, apparently analyzed the data without accounting for that correlation, which may have contributed to the “non-significant” statistical results despite the figures included with the study showing clear evidence for an effect. Luckily, the P-values in that study were still below the P < 0.37 limit and, thus, contribute to the overall significance when summarized by the FCPT.

High Fat vs High CHO

A recent study [17] with inconsistent results assessed the effect of αCD when added to a high-fat (rather than high-CHO) meal, and, consequently, blood glucose levels did not increase to levels comparable to high-CHO meals. Instead, TG levels increased, and αCD exerted a beneficial effect by reducing the increase in serum TG.

The last point relates to another important issue. With high-fat meals, αCD may have other benefits. In the study with the low-CHO/high-fat meal [17], excluded from this meta-analysis for that reason, “consumption of α-CD with a fat-containing meal was associated with a significant reduction in postprandial TG responses” [17]. This significant result was supported by the potentially “nonsignificant trend” in the study with a fat-containing meal [21].

Hence, the overall benefit of αCD is not restricted to reducing the PPG response seen after CHO-rich meals. With fat-rich meals [17], it is blood lipids (including TG) that increase, and αCD reduces this response, instead of the glucose response after a CHO-rich meal. In fact, much of the effect of αCD helping obese people to lose weight may be related to αCD reducing lipid, rather than glucose response, and a benefit αCD helping with controlling body weight has been consistently demonstrated in several clinical trials: (1) Grunberger et al. (2007) [16] and Jen (2013) [1] showed that in obese people with type 2 diabetes age >30 yr and 57.5±9, respectively, "αCD reduced body weight,” although this was “only significant after adjusting for energy intake.” (2) Comerford et al. (2011) [15] showed that in overweight people aged 41±13.6 yr, αCD “significantly decreases body weight and [low-density lipoprotein] LDL.” (3) Amar et al. (2016) [12] showed that in healthy subjects aged 34±12.4, αCD reduced small-low-density lipoprotein (LDL) by 10% (P < 0.045) and, consistent with the results of this meta-analysis, reduced insulin resistance by 11% (P < 0.04).

Finally, the FDA noted that there was no clear relationship between postprandial insulin and glucose. A lack of such a relationship, however, can, in fact, be an advantage because it shows that αCD does not lower glucose by increasing insulin production and, thus, has a desirable effect on blood glucose even in people whose insulin production is impaired.

The weaknesses of this meta-analysis include the lack of subject-level data and often even of exact P-values. Another shortcoming is that the majority of studies were conducted on young adults while the people who could potentially benefit the most are seniors. More studies in older populations are urgently needed.

## Conclusions

This meta-analysis is based on five published clinical trials on the efficacy of αCD to reduce postprandial glucose excursions after a high-CHO meal. There were strong indications that the published P-values suffered from loss of power due to errors in the statistical methodology, including the cross-over design not being reflected in the statistical method used. Still, after standardizing the dose of αCD to 5 g αCD / 50 grams of starch and excluding two additional publications, where the majority of calories came from fat, a formal meta-analysis using Fisher’s combined probability test confirmed the European Commission's (EC's) 2013 authorized health claim that when taken with a high-CHO meal "at least 5 g of the dietary fiber αCD per 50 g of carbohydrates reduce PPG excursions."

All five studies included in the meta-analysis also reported postprandial insulin levels. Interestingly, postprandial insulin levels were typically lower (albeit often not significantly lower) when the meal was taken with αCD. Hence, the beneficial effect of αCD does not require an increase in insulin production, suggesting that even people with insulin resistance might benefit from αCD.
